# Facile synthesis of polyoxometalate supported on magnetic graphene oxide as a hybrid catalyst for efficient oxidation of aldehydes

**DOI:** 10.1038/s41598-022-21991-x

**Published:** 2022-11-02

**Authors:** Ali Zarnegaryan

**Affiliations:** Department of Chemistry, Yasouj University, Yasouj, 75918-74831 Iran

**Keywords:** Catalysis, Organic chemistry, Chemistry

## Abstract

In the present study, Anderson-type polyoxometalate [N(C_4_H_9_)_4_] [FeMo_6_O_18_(OH)_6_] (FeMo_6_) was immobilized on amino-modified magnetic graphene oxide and employed as a new hybrid catalyst in oxidation of aldehydes to carboxylic acids. The synthesized hybrid catalyst Fe_3_O_4_/GO/[FeMo_6_] was characterized using thermogravimetric analysis (TGA), scanning electron microscopies (SEM), Fourier transform infrared (FT-IR), vibrating sample magnetometry (VSM), energy-dispersive X‐ray analysis (EDX), Raman spectroscopy and inductively coupled plasma atomic emission spectroscopy (ICP-OES). The results indicated that our catalyst was quite active in oxidizing the aldehydes to their corresponding carboxylic acids in the presence of hydrogen peroxide. The synthesized catalyst can be easily separated from the reaction medium and reused for six consecutive runs without a significant reduction in reaction efficiency.

## Introduction

The oxidation of aldehydes to produce carboxylic acids is an important transformation in the chemical industry^1^. Aldehydes are widely used as intermediates for the production of perfumes, agrochemicals, cosmetics, and pharmaceuticals^2^. As part of the growing tendency towards green chemistry, current research in this area has focused on the development of environmentally benign catalytic processes involving the use hydrogen peroxide or molecular oxygen as an oxidant^3^. Work towards the use of H_2_O_2_ assisted methods, in particular, has attracted considerable interest because this oxidant is providing a high active oxygen content, generates water as its sole byproduct, and readily available^4^.

Polyoxometalates (POMs) are a family of inorganic metal- oxides with diverse, and very well-defined structures^5–7^. The intriguing properties of polyoxometalates include tunable redox potential, high thermal stability, strong Brønsted acidity, photoresponse or electrical sensitivity, and inherent resistance to oxidative decomposition with a broad domain of applications in catalysis^8–13^. POMs have been used to synthesize and stabilize with regard to their highly negative charges. Anderson POMs are unique among polyoxometalates because they are composed of a single metal atom supported by a polytungstate or polymolybdate^14,15^. The POMs consist of edge-sharing metal heteroatom octahedrons (XO_6_) with six protons and six-edge sharing MO_6_ octahedral around a central^16^. The related hydroxy groups or these protons can be replaced by the organic ligands to yield a hybrid polyoxometalate. POMs can be supported on inert and high surface area materials. The resulting synergistic effects with the organic and inorganic moieties often offer extraordinarily improved performances in other research fields^17–25^.

Graphene oxide (GO) sheets are an appealing class of microporous materials with well-organized structures^26^. Graphene oxide, a new type of carbon nanomaterial composed of a monolayer of sp^2^ carbon atoms, is prominent owing to its exclusive chemical and physical properties^27^. GO is a fascinating material for catalytic applications due to its unique properties, such as high mechanical properties and high surface area^28–30^. It provides a template for immobilization of inorganic and organic species like catalysts and synergistic interactions between them; these and graphene oxide can result in improved yields. GO is an exclusive candidate for a polyoxometalate support material to overcome challenges of the high solubility of POMs, low surface area, and hybrid catalyst^31–34^. On the other hand, decorating magnetic Fe_3_O_4_ nanoparticles on GO will impart a desirable magnetic property to the graphene oxide, making the composite hopeful for numerous fields such as environment and catalysis^35–37^. Magnetic nano-catalysts are easy and efficiently removed from the resulting mixture with a magnetic nano-catalyst, and they have emerged as ideal catalysts^38^.In addition, the synergistic effects between magnetic nanoparticles and graphene oxide can appropriately prevent the re-aggregation of magnetic nanoparticles and the possible stacking of individual graphene sheets^39^. Therefore, magnetic graphene oxide would be a beneficial polyoxometalate-based catalysts support. However, by direct loading of polyoxometalates onto the supports, the serious problem of the wastage of the active species and leaching is unavoidable. A viable strategy to overcome the above obstacle could be the modification of support.

**Figure 1:**
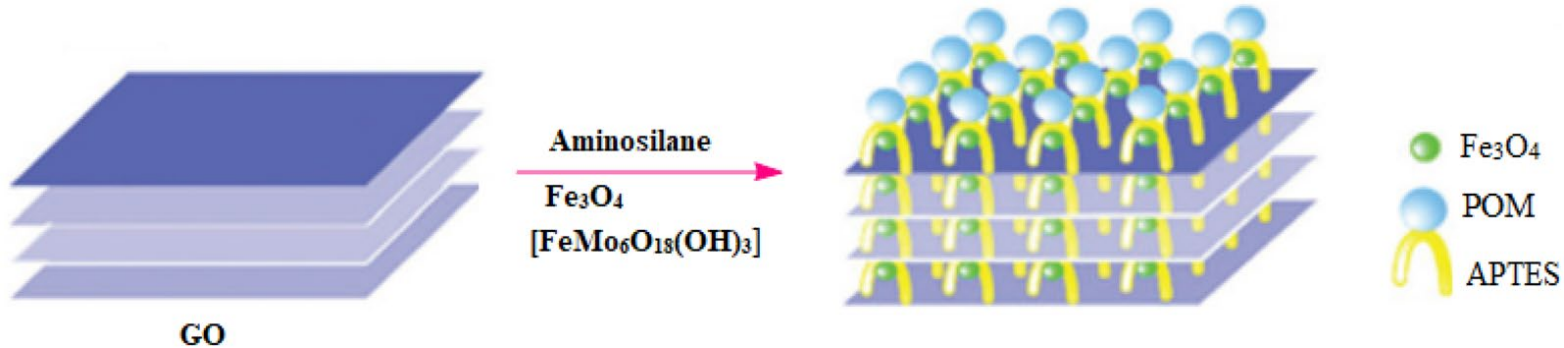
Synthetic route to the Fe_3_O_4_/GO/[FeMo_6_] hybrid catalyst.

POMs combined with graphene oxide have proved to have good performance for the oxidation of aldehydes. Due to the synergetic effect and benefitting from the potential catalytic sites offered by polyoxometalate, nanoparticles (NPs), and graphene oxide, outperform the corresponding homogeneous catalysts. A wide variety of catalytic systems have been developed for the oxidation of aldehydes to carboxylic acids, including metal complexes^40^, metal oxides^41^, and polyoxometalates (POMs)^42^. POMs combine high reactivity and stability in oxidation catalysis. Some of the recent reports on polyoxometalate-based catalytic systems for oxidation of alcohols, aldehydes, olefins and oxidative carbon–carbon bond cleavage of 1,2-diols to carboxylic acids / ketones with hydrogen peroxide^43–51^. Herein we report the design of a new nanocomposite amine-functionalized graphene oxide immobilized with Anderson-type POM clusters (Fig. 1), which feature a well-defined structure and stability and show promising catalytic performance as a catalyst for oxidation of various aldehydes.

## Experimental

### Synthesis of [N(C_4_H_9_)_4_]^3^[FeMo_6_O_18_(OH)_6_]0.7H_2_O [FeMo_6_]

[N(C_4_H_9_)_4_][FeMo_6_] was synthesized according to a published method^52^, with suitable modifications: ammonium molybdate (7.95 g) was dissolved in H_2_O (125 mL) and then it was heated to 100 °C. Iron (III) sulfate (1.9 g) was dissolved in H_2_O (60 mL), which was stirring, was slowly added to the solution by stirring. The mixture was continued stirring for 1 h after complete addition, and then the crude N(C_4_H_9_)_4_ salt filtrate was obtained from the solution.

### Synthesis of the Fe_3_O_4_/GO

Graphene oxide was synthesized according to a published method^53^. GO/Fe_3_O_4_ was prepared according to the reported literature^54–57^. In a typical preparation, 0.8 g GO was dispersed in 180 mL deionized water for an hour to obtain an aqueous suspension of GO nanoparticles. 2 mmol FeCl_3_.6 H_2_O and 1 mmol FeCl_2_.4 H_2_O were dissolved in 80 mL deionized water by stirring and under N_2_ atmosphere for 30 min at 80 °C. GO suspension was added gradually to this solution. Finally, the black precipitate was separated by a strong magnet, washed with deionized water and ethanol, and then vacuum-dried at 80 °C overnight.

### Synthesis of amino-saline functionalized magnetic graphene oxide (Fe_3_O_4_/GO-NH_2_)

Aminopropyltrimethoxysilane was grafted on graphene oxide targeting their hydroxyl and carboxyl groups^30^. In order to functionalize GO nano-sheets with amine groups, 1 g Fe_3_O_4_/GO was dispersed in 80 mL water and sonicated for 40 min. Then 250 mL ethanol and 10 mL 3-aminoprpyltriethoxysilane (APTES) were added and mechanically stirred for 50 min. Then, the mixture was refluxed overnight at 80 ℃. The obtained solid was washed with absolute ethanol to remove unreacted species.

### Synthesis of the Fe_3_O_4_/GO/[FeMo_6_]

To immobilize [FeMo_6_O_18_(OH)_6_] on the Fe_3_O_4_/GO, a stirred solution of Fe_3_O_4_/GO-NH_2_ (0.20 g) in CH_3_CN solvent (10 mL) at 70 ℃, a solution of FeMo_6_ (0.12 g) in CH_3_CN (10 mL) was added dropwise. The reaction mixture was vigorously stirred at reflux for 20 h. The composite material was then isolated by vacuum filtration, and it subsequently sonicated in CH_3_CN solvent for 6 h. Finally, the material was washed entirely with C_2_H_5_OH two times and it was dried at 45 ℃. The amount of Fe and Mo were measured by inductively coupled plasma atomic emission spectroscopy (ICP-OES). The ICP-OES results of Fe_3_O_4_/GO/[FeMo_6_] showed that the iron and molybdenum contents of the nanocomposite were 19.51 and 27.4%, respectively.

### Catalytic tests

Fe_3_O_4_/GO/[FeMo_6_] (2.69 mg), aldehyde (2.0 mmol), H_2_O_2_ (5.0 mmol) using Na_2_CO_3_ (15.9 mg), and ethanol (12 mL) were added to a round-bottom flask. The mixture was stirred at 40 ℃, and the reaction progress was determined using thin-layer chromatography (TLC). The solid catalyst was isolated using a magnetic field and was used in subsequent reactions.

## Results and discussion

**Figure 2:**
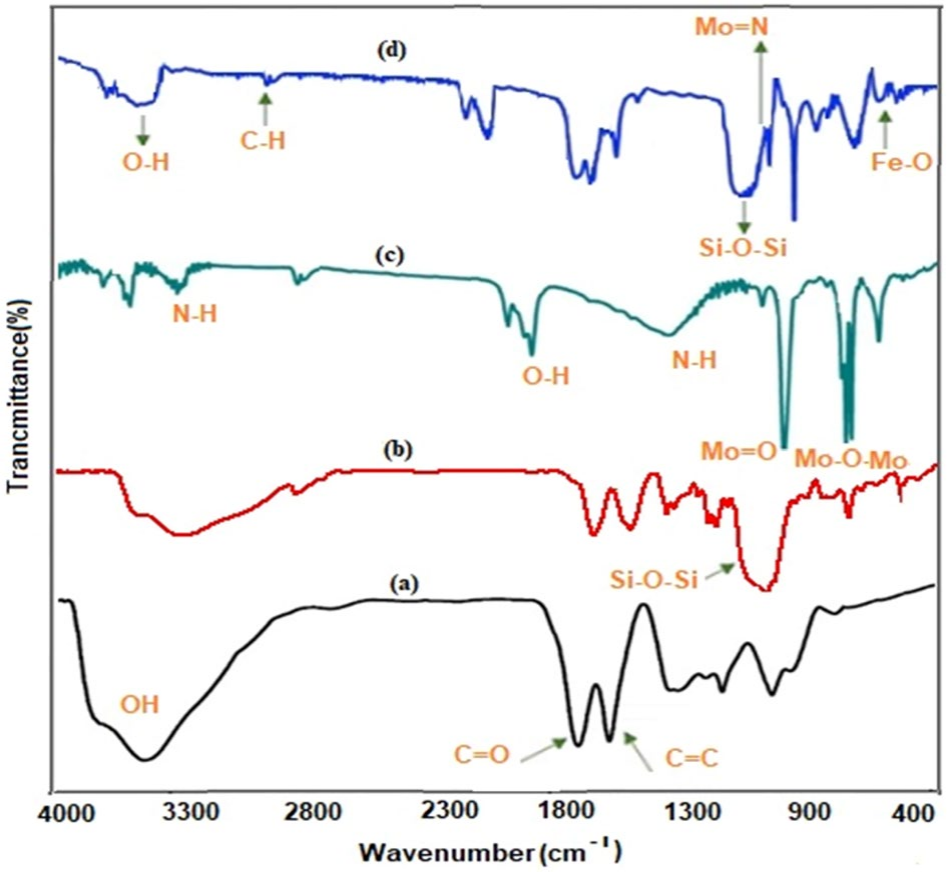
FT-IR spectra of (**a**) GO, (**b**) Fe_3_O_4_/GO-NH_2_, (**c**) POM, and (**d**) Fe_3_O_4_/GO/[FeMo_6_].

Fourier transform infrared (FT-IR) analysis is utilized for the chemical structure of POM, GO, and Fe_3_O_4_/GO/[FeMo_6_] catalyst (Fig. 2). The spectrum of GO exhibits characteristic bands at 3381, 1724, 1620, 1221, and 1056 cm^–1^ (Fig. 2a) corresponding to the attendance of O–H, C=O, C=C, C–O–C, and C–O, respectively^58^. Peaks at 1042 and 1131.7 cm^–1^ (Fig. 2b) illustrate the presence of Si–O–C and Si–O–Si bonds, respectively. The characteristic peak of Anderson-type POM (Fig. 2c) appeared at 949 (Mo=O) and 648 (Mo–O–Mo) cm^–1^^48,59^. However, the nano-catalyst showed peaks at 945, and 671 cm^–1^ (Fig. 2c). The signals at 2848 and 2947 cm^–1^ are attributed to the vibrations of the C-H bonds (Fig. 2d). The attendance of SiO_2_ and Fe_3_O_4_ in the nano-catalyst was illustrated with the observation of vibration bands 3417, 1063, and 589 cm^−1^ assigned to O–H vibration Si–O–Si, and Fe–O. Thus, it is evident that the Fe_3_O_4_ nano-particles and FeMo_6_ cluster units are in attendance in the obtained nano-catalyst.

**Figure 3:**
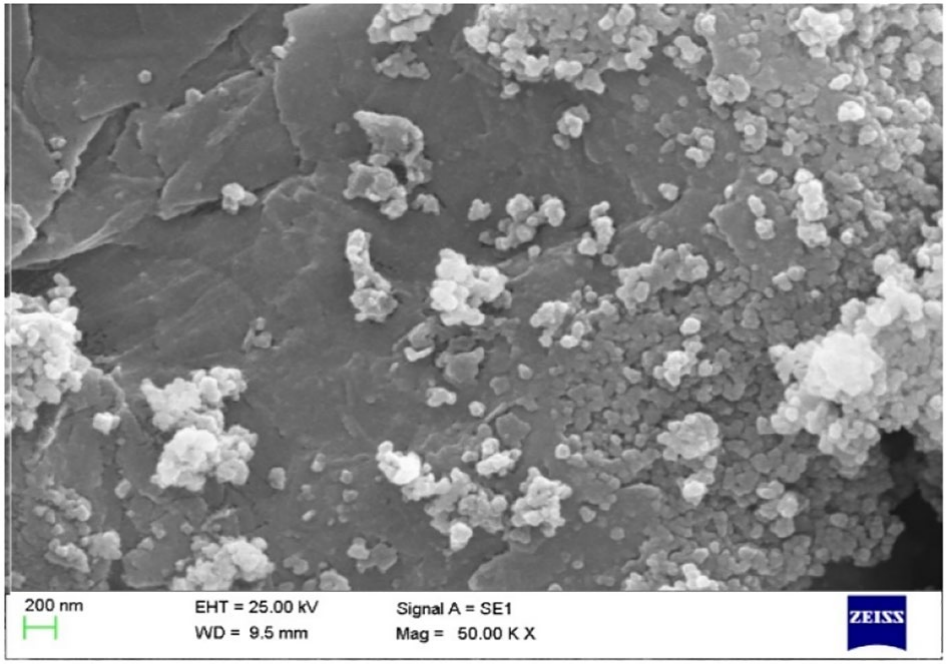
SEM of the Fe_3_O_4_/GO/[FeMo_6_].

**Figure 4:**
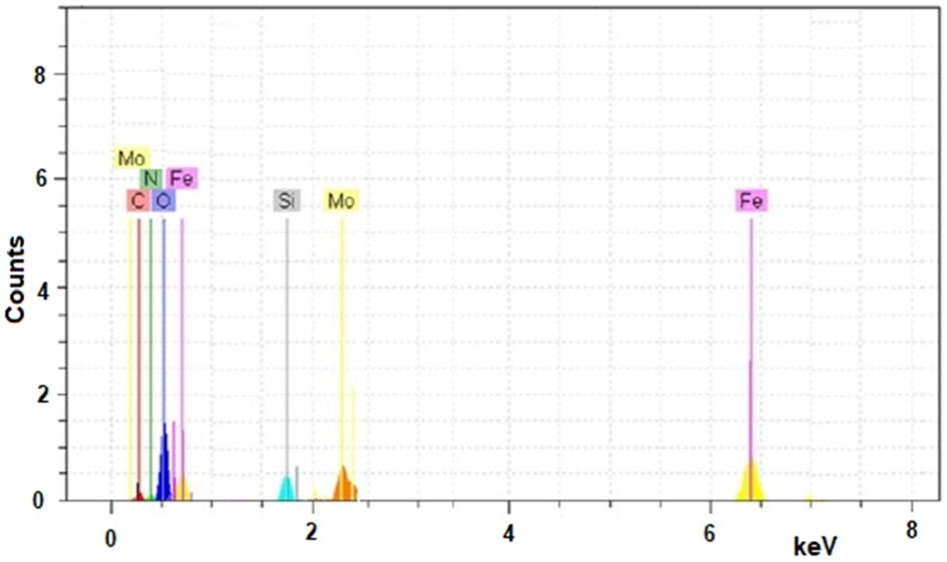
EDX of the Fe_3_O_4_/GO/[FeMo_6_].

Figure 3 shows the scanning electron microscopy (SEM) image of Fe_3_O_4_/GO/[FeMo_6_]. Images of Fe_3_O_4_/GO/[FeMo_6_] catalyst revealed a sparse distribution of individual Fe_3_O_4_ nanoparticles and large agglomerates on graphene sheets, suggesting that GO sheets can act as excellent supports for the embedding of the POMs and the Fe_3_O_4_. The chemical composition of Fe_3_O_4_/GO/[FeMo_6_], studied by energy-dispersive X-ray spectroscopy (EDX), revealed the attendance of Fe, Mo, C, O, Si and N elements in the sample, confirming good immobilization of POM and Fe_3_O_4_ species onto graphene sheets (Fig. 4).

**Figure 5:**
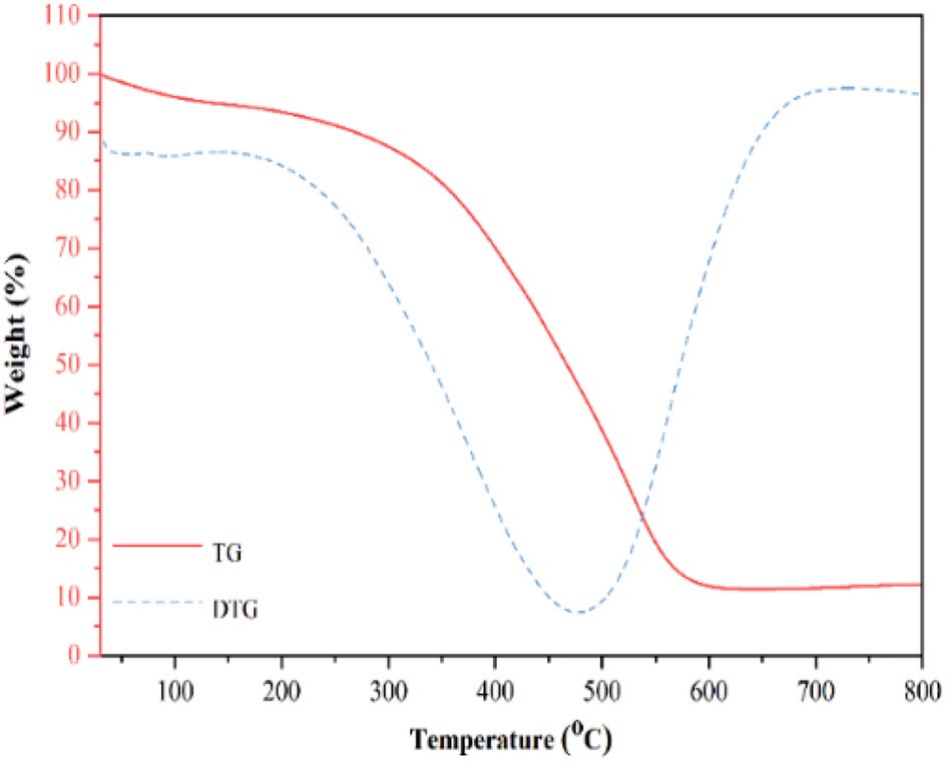
TG analysis of the Fe_3_O_4_/GO/[FeMo_6_].

The thermal behavior of catalyst was investigated by thermal gravimetric analysis (TGA), as illustrated in Fig. 5. The thermal gravimetric analysis plot of the catalyst demonstrates a three-step mass loss around 25–650 °C. The first 4.62% weight loss at 100 °C can be accounted for solvent associated with the compound^60^. The weight loss starting at 220 °C of 48.20% corresponds to the loss of the organic cations and the ligands^61^. At about 590 °C, the decomposition of the metal oxide starts.

**Figure 6:**
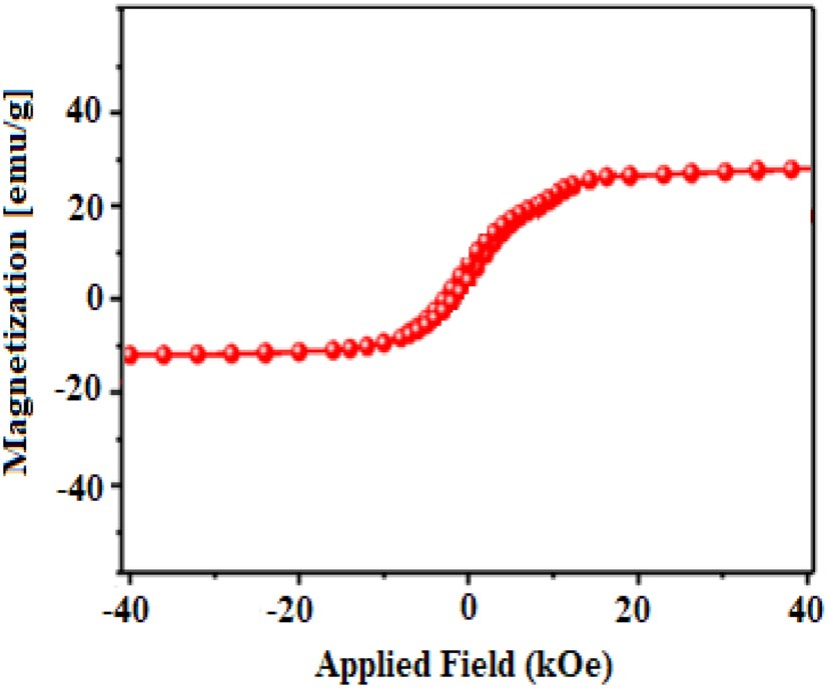
VSM analysis of the Fe_3_O_4_/GO/[FeMo_6_] nanocatalyst.

The magnetic behavior of the obtained Fe_3_O_4_/GO/[FeMo_6_] catalyst is demonstrated in Fig. 6. This shows magnetization of about 21.6 emu/g and confirms an excellent superparamagnetic behavior of the material that is a fundamental characteristic, especially for catalytic processes.

**Figure 7:**
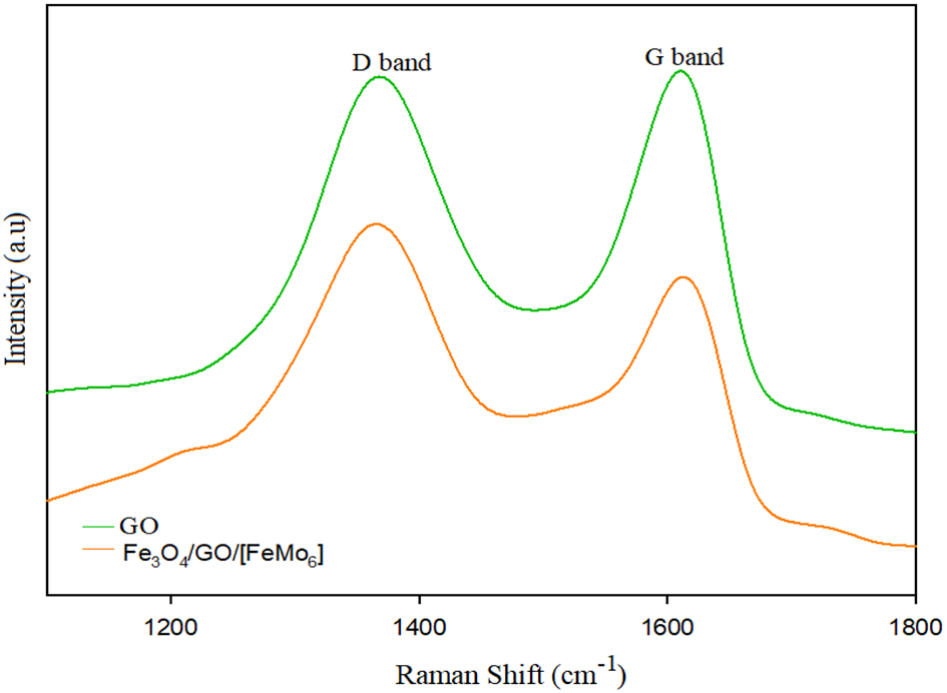
Raman spectra obtained on the GO and Fe_3_O_4_/GO/[FeMo_6_].

The significance of the interaction between GO and [FeMo_6_] can be analyzed by the changes in the carbon framework by Raman spectra. Raman spectra with characteristic G (vibration of sp^2^ carbon atoms) and D (vibration of the sp^3^ carbon atoms), bands sensitive to disorder, carbon grain size, and defects, have extensively been used to characterize carbon materials^62^. The Raman spectra of GO and Fe_3_O_4_/GO/[FeMo_6_] are shown in Fig. 7. The position of the D band is almost the same before and after the chemical modifications. However, the G band shifts from 1609 for GO to 1613 cm^−1^ for Fe_3_O_4_/GO/[FeMo_6_] catalyst. This may be attributed to the attachment of the polyoxometalate on GO, causing an increased defect density in graphene sheets^62^.

### Catalytic activity of Fe_3_O_4_/GO/[FeMo_6_]

The catalytic activity of the prepared Fe_3_O_4_/GO/[FeMo_6_] nano-catalyst has been tested as a catalyst for the synthesis of carboxylic acids using the oxidation of aldehyde. The data for the optimization of reaction conditions, using aldehyde (2.0 mmol), additive (0.3 equiv), and H_2_O_2_ (5 mmol) as the oxygen donor in the presence of Fe_3_O_4_/GO/[FeMo_6_] catalyst (0. 3 mol%) using various solvents, are given in Table 1. The reaction was carried out at 45 °C using various additives (Table 1). It was also found that the Fe_3_O_4_/GO/[FeMo_6_] catalyst is necessary for the synthesis of carboxylic acids owing to no product appearing in the absence of the catalyst (entry 1). The effect of the additives was investigated. When Na_2_CO_3_ was added to the reaction, carboxylic acid was synthesized with a yield of 98% (entry 2), while the addition of NaHCO_3_ dropped the yield of carboxylic acid to 67% (entry 3). Using Na_2_SO_3_ further dropped the yield to 57% (entry 4), while Na_2_SO_4_ denoted the lowest yield of 23% (entry 5). The basic additives, Et_3_N and CH_3_COONa, gave carboxylic acid in 85 and 79% yields (entries 6 and 7), respectively. Additives with a neutral salt, like sodium chloride, gave moderate yields of the generated product (entry 8).

**Table 1:** The effects of conditions.


Entry	Additive	T (°C)	Mol (%) cat	Yield (%)
1	Na_2_CO_3_	45		–
2	**Na**_**2**_**CO**_**3**_	45	0.3	**98**
3	NaHCO_3_	45	0.3	67
4	Na_2_SO_3_	45	0.3	57
5	Na_2_SO_4_	45	0.3	23
6	Et_3_N	45	0.3	85
7	CH_3_COONa	45	0.3	79
8	NaCl	45	0.3	68
9	–	45	0.3	27
10	Na_2_CO_3_	45	0.6	96
11	Na_2_CO_3_	45	0.4	97
12	Na_2_CO_3_	45	0.2	94
13	Na_2_CO_3_	25	0.3	87
14	Na_2_CO_3_	60	0.3	92
15	Na_2_CO_3_	70	0.3	89

Significant values are in bold.

Entries 10–12 in Table 1 indicate the effect of the nano-catalyst loading, and entries 13–15 in Table 1 show the effect of reaction temperature on the reaction efficiency. Investigation of the results in Table 1 indicated a solvent-dependent product for the formation of carboxylic acid. The best efficiency (98%) was achieved at 45 °C in EtOH.

Afterward, a series of carboxylic acid derivatives were synthesized in the reaction of various functionalized aldehydes at optimized reaction conditions of catalyst (Table 2). To our satisfaction, good yields were obtained with various aldehydes that we studied. It is important to note that for all substrates, Fe_3_O_4_/GO/[FeMo_6_] catalyst was obtained, confirming the high efficiency of the designed nano-catalyst to synthesize a wide range of carboxylic acid derivatives. Aromatic aldehydes bearing R groups such as electron-withdrawing groups and electron-donating groups obtained a high yield. Various aliphatic aldehydes were also tested and gave the corresponding products in excellent yields.

**Table 2:**
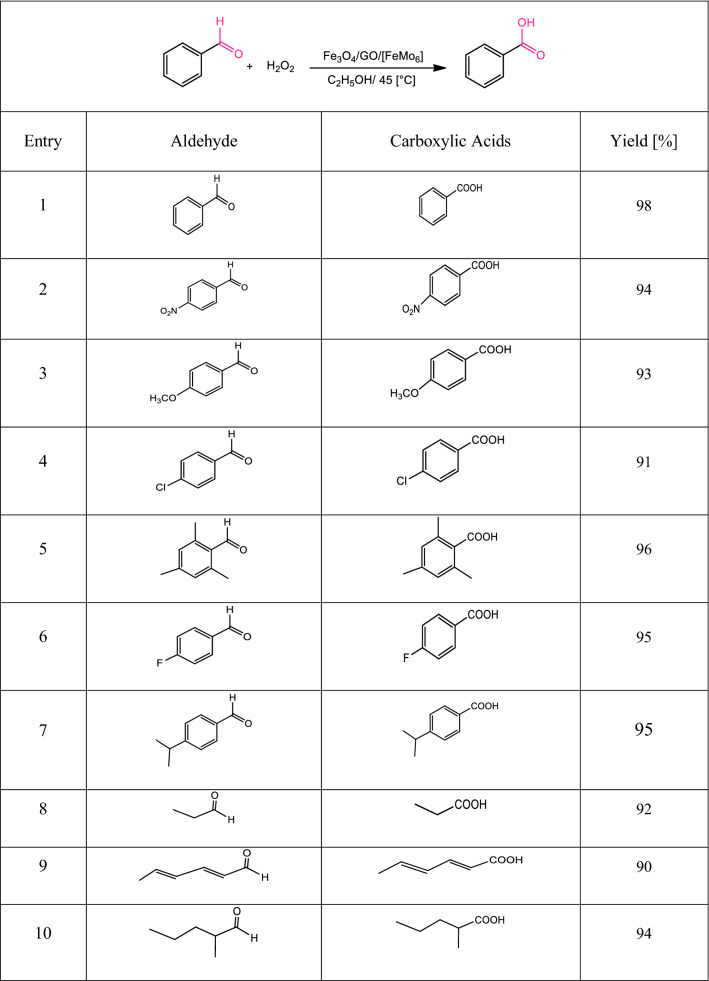
Investigation of substrate scope.^[a]^

^[a]^Reaction conditions: catalyst (0.3 mol%), aldehyde (2 mmol), H_2_O_2_ (5 mL), additive (0.3 equiv), C_2_H_5_OH (12 mL).

### Recycling of the Fe_3_O_4_/GO/[FeMo_6_]

**Figure 8:**
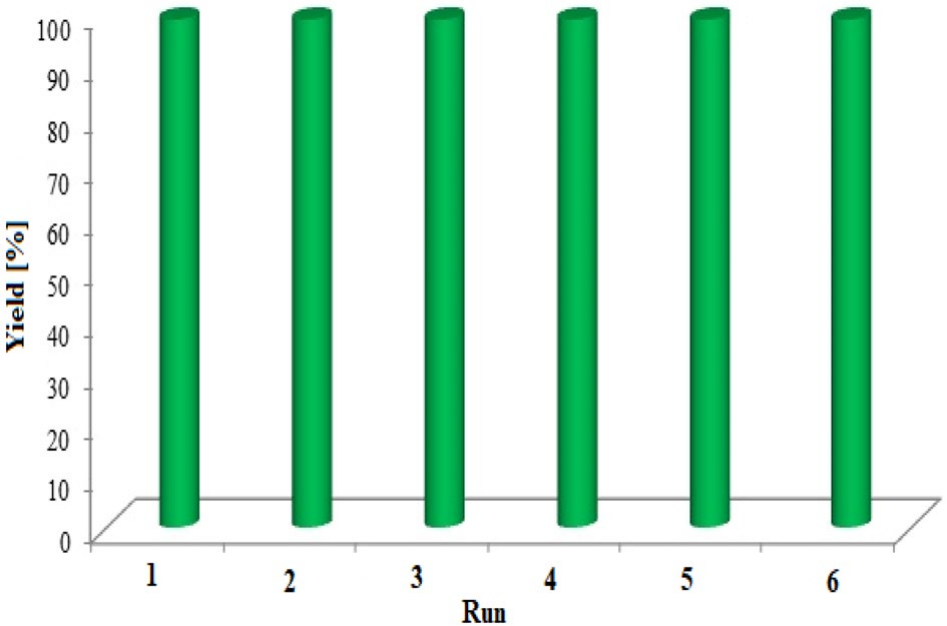
Recycling experiments of the Fe_3_O_4_/GO/[FeMo_6_].

To further determine the stability of the Fe_3_O_4_/GO/ [FeMo_6_] nano-catalyst, the catalytic activity of the recovered catalyst was evaluated in the synthesis of carboxylic acids under similar conditions, and the results are given in Fig. 8. The results demonstrated that after six consecutive runs, the catalytic activity has no significant changes and that the catalyst is active and stable after recycling.

**Figure 9:**
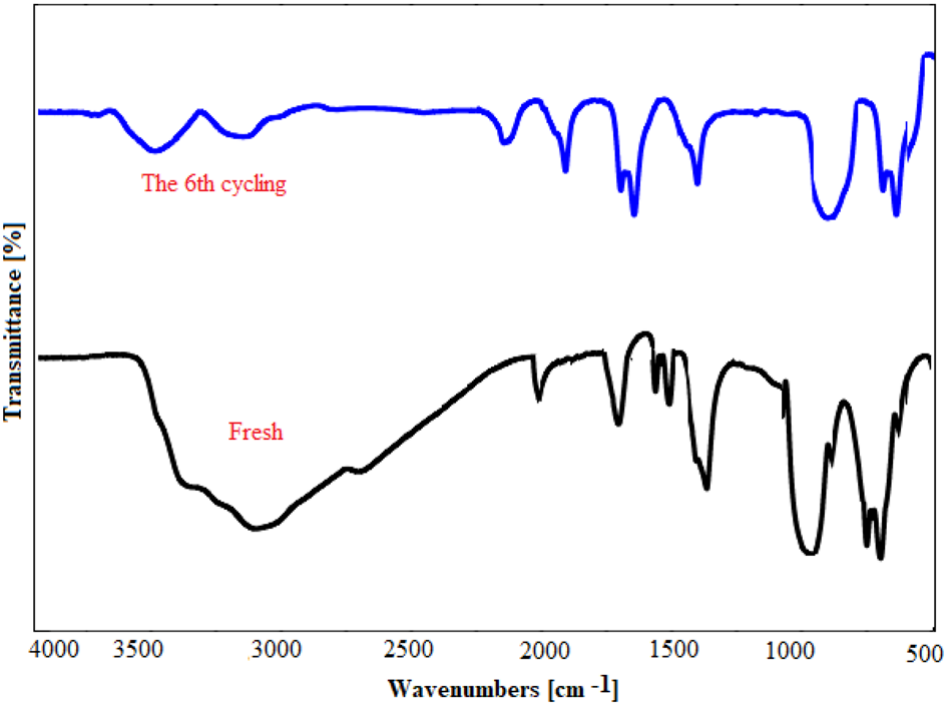
The FT-IR spectra of the catalyst before and after the reaction.

To confirm the high stability of the nano-catalyst and its associated performance, the structure of the catalyst was further investigated using FT-IR spectra (Fig. 9). FT-IR pattern of the recovered catalyst was the same as the FT-IR spectrum of the fresh catalyst, proving the high chemical stability of the Fe_3_O_4_/GO/ [FeMo_6_] nano-catalyst under applied conditions.

### Catalytic mechanism

**Figure 10:**
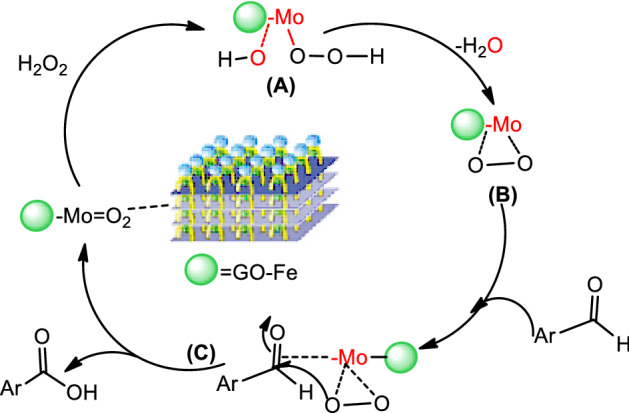
Proposed mechanism of oxidation process by Fe_3_O_4_/GO/[FeMo_6_].

Based on some previous reports^63–65^ the possible catalytic mechanism of Fe_3_O_4_/GO/[FeMo_6_] was illustrated in Fig. 10. The peroxide species could be formed via nucleophilic attack of H_2_O_2_ on the surface of the nano-catalyst (A). The immobilized [FeMo_6_] species on the Fe_3_O_4_/GO surface are converted to polyoxoperoxo complexes (B). Polyoxoperoxo reacts with aldehydes molecules in step (C), and corresponding carboxylic acids is generated. Therefore, the reaction rate of peroxide species is a key factor which affects the oxidation efficiency of aldehydes.

The catalytic efficiency of our constructed catalyst in the preparation of carboxylic acids has been compared with the previously reported methods in reaction time, temperature, and yields of products with aldehydes (Table 3).

**Table 3:** A comparison study between the performance of Fe_3_O_4_/GO/[FeMo_6_] and former catalysts in the synthesis of carboxylic acids.

Entry	Catalyst	Conditions	Time (h)	Yield (%)	Ref
1	MOF-Zn-NHC (15 mg)	H_2_O, Reflux	3.5	80	^66^
2	CuO@HPS (4 mol%)	H_2_O, 75 °C	20	80	^41^
3	bis-NHCs (0.05 mmol)	DMSO, 60 °C	36	91	^67^
4	Cu (OAc)_2_/by (10.0 mol%)	H_2_O, 50 °C	12	68	^68^
5	Fe_3_O_4_/GO/ [FeMo_6_] (0.3 mol%)	EtOH, 45 °C	2	98	This work

## Conclusion

In summary, a new, efficient and recyclable hybrid catalyst was prepared successfully and used for the oxidation of various aldehydes with H_2_O_2_ as an oxidant. The catalyst, Fe_3_O_4_/GO/[FeMo_6_], made up of a Fe_3_O_4_ nanoparticle immobilized on graphene oxide-supported polyoxometalate, was easily synthesized. With this nanocatalyst, various structurally diverse aldehydes were successfully transformed into the corresponding carboxylic acids in excellent yields. Moreover, the recyclability test exhibited that it could be reused for six consecutive runs without appreciable loss in catalytic efficiency. The additional advantages of the present nanocatalyst include simplicity, yield, cost, reaction time, and selectivity as compared to other catalysts available in the literature for the same organic transformation. Furthermore, easy catalyst recovery, faster synthesis, recyclability and inexpensive reactants make this methodology a potential candidate for sustainable synthesis.

## Data Availability

The datasets used and, or analyzed during the current study are available from the corresponding author upon reasonable request.
